# DEMENTIA-RELATED STIGMA IS ALIVE AND NOT WELL AMONG UNREGULATED HOME CARE WORKERS

**DOI:** 10.1093/geroni/igac059.1604

**Published:** 2022-12-20

**Authors:** Marie Savundranayagam, Shalane Basque, Audrey Gruneberg

**Affiliations:** Western University, London, Ontario, Canada; Western University, London, Ontario, Canada; Western University, London, Ontario, Canada

## Abstract

Dementia-related stigma is pervasive despite the growing rates of dementia globally. The social cognitive model of stigma contends that stigma is created and maintained through negative stereotypes, prejudice, and discrimination towards people who belong to a group. Research on dementia-related stigma highlights the prevalence of stigmatizing views among formal care providers. However, little is known about how stigma is created and maintained by the ways in which frontline care providers talk about persons living with dementia. Accordingly, this study aimed to identify ways in which stigmatizing language is used by home care workers when describing routine care interactions with clients living with dementia. Semi-structured interviews were conducted with 30 unregulated home care workers, who shared their experiences caring for clients with dementia. We used conventional content analysis to identify themes related to dementia-related stereotypes, prejudice, and discrimination. Under stereotypes, persons with dementia were portrayed as objects, infants, not engaging, and cognitively and behaviorally unstable. Under prejudice, persons with dementia invoked pity, fear, disdain, and emotional fatigue. Under discrimination, participants shared experiences of excluding/ignoring, controlling, and using patronizing communication with persons living with dementia. Uncovering common examples of stigmatizing language offers opportunities to train home care workers to use person-centered communication. It is noteworthy that stigmatizing language emerged, when not asked about directly. Our findings underscore the persistence of dementia-related stigma and the need for training to eliminate stigma.

